# “Fistula Zero” Project After Total Laryngectomy: The Candiolo Cancer Institute Experience

**DOI:** 10.3389/fonc.2021.690703

**Published:** 2021-06-22

**Authors:** Erika Crosetti, Giulia Arrigoni, Andrea Elio Sprio, Giovanni Succo

**Affiliations:** ^1^ Head and Neck Oncology Unit, Candiolo Cancer Institute, Fondazione del Piemonte per l’Oncologia - IRCCS (FPO-IRCCS), Candiolo, Italy; ^2^ Department of Biomedical and Clinic Sciences, University of Turin, Orbassano, Italy; ^3^ Department of Oncology, University of Turin, Orbassano, Italy

**Keywords:** laryngeal cancer, total laryngectomy, pharyngocutaneous fistula, complications, Montgomery tube, bypass tube, pedicled flap, head and neck cancer

## Abstract

**Objectives:**

Pharyngocutaneous fistula (PCF) is a troublesome complication after total laryngectomy. The “Fistula zero” project aims to reduce the number of PCF by following a detailed protocol based on three fundamental key points.

**Materials and Methods:**

The Fistula zero project included 77 patients who underwent total laryngectomy in the period from January 2019 to December 2020. The protocol consisted of three main aspects: the systematic placement of a Har-El salivary bypass tube, the continuous horizontal watertight pharyngeal suture using a barbed suture, onlay insetting of a pedicled flap in pre-treated patients.

**Results:**

One case of PCF (1.3%) and three small blind fistulas (3.9%) were observed in this series. The mean length of hospitalization was 18 days.

**Conclusion:**

Pharyngocutaneous fistula (PCF) prolongs hospitalization and delays adjuvant treatments. Thanks to a strict adherence to the protocol, it was possible to reduce PCF rates, avoiding lengthy hospitalization and additional surgical procedures.

## Introduction

Total laryngectomy (TL) is considered the gold standard surgical treatment for advanced laryngeal cancer. Surgery can be performed as primary treatment or as salvage treatment after failure of previous surgical or non-surgical protocols.

Pharyngocutaneous fistula (PCF) is a common complication after TL, with an incidence ranging from 3% to 65% (1–4) [9–25% after primary surgery, 30–70% after salvage laryngectomy (5)]. When PCFs occur after total laryngectomy, several challenging consequences have to be expected: delays for adjuvant treatments, frequent need for revision surgery, an increase in the length of hospitalization, delays in the rehabilitation process leading up to oral food intake, reduction in quality of life, and higher costs (both social and economic).

Many studies have focused on the risk factors associated with PCF (6, 7) and many surgical strategies have been proposed to reduce its incidence (3, 5, 8, 9). Of these, one is represented by the routine placement of a salivary bypass tube. Even though some interesting results related to this strategy are reported in the literature, robust evidence is still lacking (10, 11).

The main aim of this study is to present the results of the project, titled “Fistula zero after total laryngectomy.”

## Materials and Methods

In total, 77 consecutive patients underwent primary/salvage TL at the Head and Neck Oncological Unit of the FPO IRCCS, Candiolo Cancer Institute, in the period from January 2019 to December 2020 and were included in this study.

All of the procedures performed were considered to be conventional in terms of technique and indications, according to the current guidelines, to the ethical standards of the Institutional and/or National Research Committee and to the 1964 Helsinki Declaration and its later amendments. Ethical review and approval were not required for this study in accordance with the national and institutional requirements. Before surgery, every patient signed a consent form for the disclosure of appropriate personal data for scientific purposes. Written informed consent was obtained from all of the patients. They all underwent the same clinical assessment during the 3 weeks before surgery, including clinical examination, nutritional status evaluation (body mass index [BMI]), biopsy/pathological examination, maxillofacial and neck MRI/CT scan, and total body PET scan. Two surgeons (GS and EC) carried out all of the procedures. Demographic data for the study population are summarized in **Table 1**.

**Table 1:** Demographic data for the 77 patients in this study.

Characteristic	No. of patients (%)
**Age, years**	
Mean	67 ± 10
Range	47–90
**Sex**	
Male	70 (90.9)
Female	7 (9.1)
**Comorbidities**	
No	15 (19.5)
1 comorbidity	18 (23.4)
2 comorbidities	30 (38.9)
≥3 comorbidities	14 (18.2)
	
ACE-27 Grade 1	39 (50)
ACE-27 Grade 2	19 (24)
ACE-27 Grade 3	4 (5)
**BMI**	
Mean	24
Range	17–31
**Pre-treatment**	
Yes	27 (35.1)
No	50 (64.9)
**Pathological status**	
pT4a	27 (35.1)
pT3	19 (24.7)
pT2	4 (5.2)
ypT4	16 (20.8)
ypT3	6 (7.8)
ypT2	5 (6.5)
**Neck dissection**	
** Ipsilateral**	30 (39)
Pre-treated	12 (17)
Primary	18 (23)
	
** Bilateral**	40 (51.9)
Pre-treated	8 (10)
Primary	32 (41)
	
**None**	
Pre-treated	7 (9.1)
**Thyroidectomy**	
Hemi-thyroidectomy	20 (26)
Total thyroidectomy	27 (35.1)
Hystmectomy	30 (38.9)
**Complications**	
PCF	1 (1.3) [not-pre treated]
Minimum extraluminal spill of barium	3 (3.9) [2 pre-treated (2.5%), 1 primary patient (1%)]

**Figure 1:**
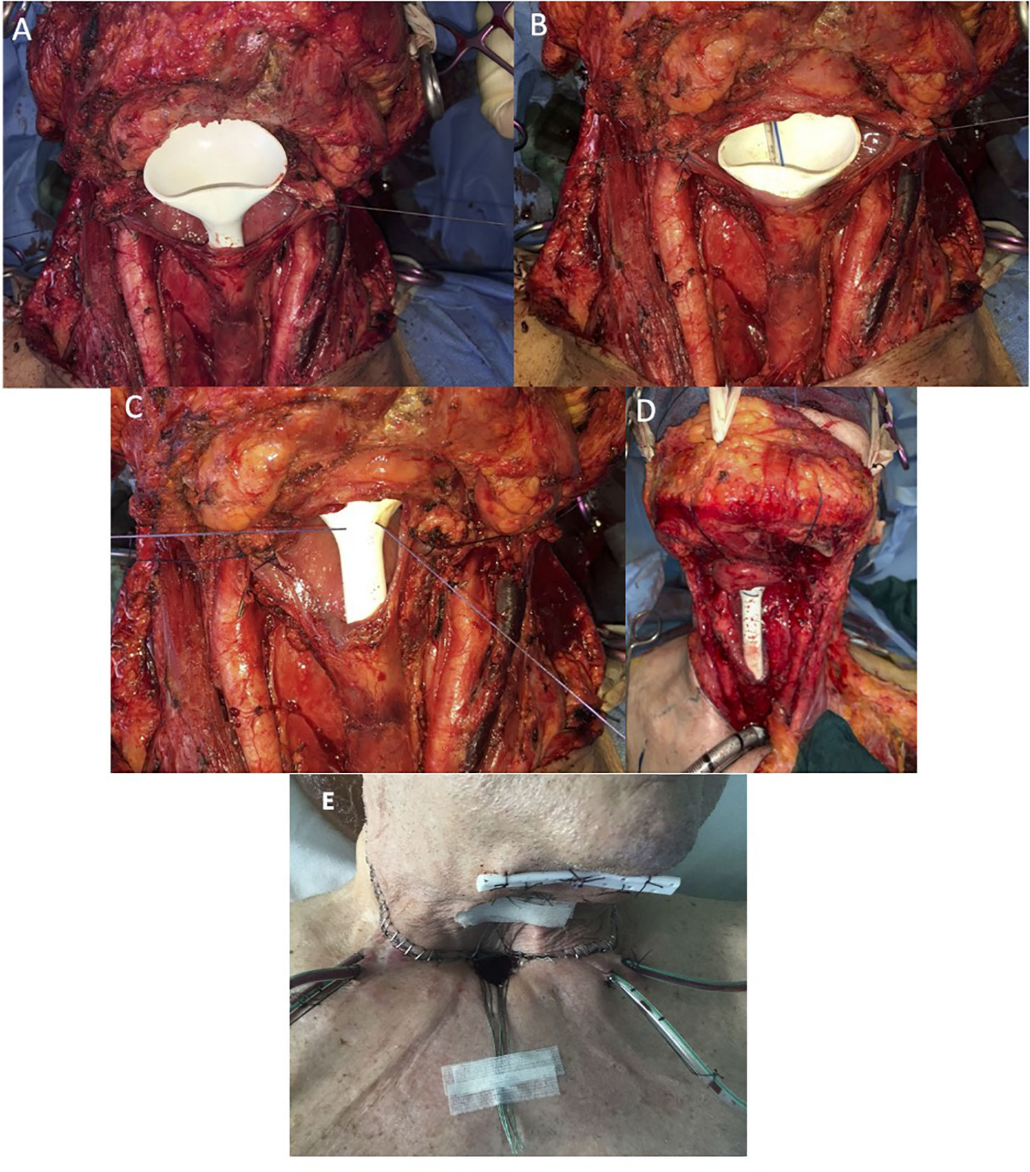
**(A)** Intraoperative positioning of salivary bypass tube. **(B)** Naso-gastric tube (NGT) insertion inside salivary tube. **(C, D)** Transcutaneous securing of salivary tube. **(E)** Salivary stent fixation on the skin.

The “fistula zero protocol” was adopted for the whole series. Following the protocol, a Har-El salivary bypass tube (Boston Medical™, Westborough, MA, USA) was placed in the neopharynx with a naso-gastric tube (NGT) positioned inside it before performing the pharyngeal closure; the bypass tube was secured by a stitch passing through the base of the tongue toward the skin, where it was knotted to prevent pressure ulcers (**Figures 1A–E**). The Har-El pharyngeal tube was chosen because of its particular funnel shape, designed to be easily anchored at the tongue base. The posterior aspect of the tube is higher while the anterior wall has a lower, flattened profile: this feature prevents it from being displaced upward into the oropharynx.

A watertight pharyngeal suture was performed using a continuous barbed suture (V-lock suture; Covidien™, Mansfield, VA, USA). Two resorbable stitches were placed at each end of the neopharynx to keep the mucosa stretched; two V-lock sutures were used to suture each hemipharynx as far as the midline. A second layer of reinforcement was then made with a third barbed suture. In pre-treated patients [radiotherapy (RT) with or without concomitant chemotherapy (CRT)], a pedicled flap (pectoralis major myofascial flap) was harvested and placed upon the pharyngeal suture.

Patients who had undergone earlier laryngeal surgical procedures [transoral laser microsurgery (TLM)/open partial horizontal laryngectomy (OPHL)] were not included in this group. This study focused on patients pre-treated with chemoradiotherapy, since such treatments may represent one of the main risk factors for postoperative fistula formation.

In every patient, a barium dynamic swallowing test with liquid and semi-liquid bolus was performed in both antero-posterior and lateral projections before commencing oral intake. The exam was performed on the 9th postoperative day in the group of patients where no reconstructive surgery was carried out, and on the 12th postoperative day in the group of patients who underwent reconstructive surgery by pectoralis major myofascial flap. In the case of absence of PCF, oral food intake started on the 10th postoperative day for patients who underwent only total laryngectomy without reconstruction, and on the 13th postoperative day for those who underwent reconstructive surgery. Oral feeding began with the salivary bypass tube in place and after 2 days, the patient was discharged. The NGT was removed on the same day as commencement of oral feeding and the salivary bypass tube was removed 1 week after the barium swallowing test (if it showed no fistulas).

When the barium swallowing test indicated the presence of a PCF/blind fistula, a compressive cervical dressing was put in place and oral feeding was delayed.

## Results

The fistula zero protocol was applied in 77 patients (70 men, 7 women) who underwent primary/salvage TL for laryngeal cancer; the median age was 67 years (range, 47–90 years).

In total, 27 patients (35.1%) were pre-treated [RT: 7 patients (9.1%), CRT: 20 patients (26.0%)]. Comorbidities were scored by applying the Adult Comorbidity Evaluation-27 Index (12). This score is composed of 12 organ system related categories and 27 subcategories; it aims to quantify a specific disease. The Overall Comorbidity Score was defined according to the highest ranked single disorder (0 = none, 1 = mild, 2 = moderate, and 3 = severe). More than two grade 2 scores gave an overall score of 3.

Nineteen patients (25%) were staged pT3: they were not amenable to OPHL or organ-sparing protocols due to important comorbidities (ACE-27 grade 2). Moreover, some of them had undergone previous chemoradiotherapeutic treatments for oropharyngeal squamous cell carcinoma/non-Hodgkin lymphoma. Four patients (5%) were very old (>80 years) and had severe comorbidities (ACE-27 grade 3): in these cases, we preferred to carry out a total laryngectomy.

The median intraoperative time for the surgical procedure was 210 min.

The salivary bypass tube demonstrated good patient-tolerability with no complications (bypass tube dislocation, migration, granulation tissue formation). The average length of hospitalization was 18 days (range, 12–40 days): 21 days for patients who underwent reconstruction and 16 days for those who did not.

PCF occurred in only one patient who had not been pre-treated (1.3%), who experienced more than three comorbidities (cardiopathy, diabetes, kidney failure, chronic obstructive pulmonary disease, ACE-27 grade 3) and who was treated by revision surgery (a pectoralis major myofascial flap was harvested and placed to cover the PCF). Three patients (3.9%), two pre-treated and one not pre-treated, developed a minimum extraluminal spill of barium (blind fistula), successfully managed with a compression dressing. In these cases, oral feeding was postponed. Donor-site complication (seroma) occurred in four pre-treated patients (5%) and was managed with a compression dressing.

## Discussion

Complications after TL are always associated with delayed adjuvant treatment, a longer hospitalization due to the delayed rehabilitation, and the need for additional postoperative surgical procedures. However, a trend toward a reduction in postoperative complications has recently been reported. In a systematic review of 522 studies (2177 patients), Sayles and Grant reported an incidence of 14.3% for pharyngocutaneous fistula in patients treated by primary laryngectomy with an increased incidence of 27.6% after salvage laryngectomy (3).

There are several risk factors related to PCF, which can be summarized as patient-related, disease-related and treatment-related. Concerning patient-related risk factors, age > 60 years, presence of > 1 comorbidity (lung disease, cardiopathy, diabetes), smoking status, nutritional status, low albumin level, and low hemoglobin level (7, 13–16) have to be considered. Disease-related risk factors are represented by advanced T stage and tumor site (supraglottic region) (17). Regarding treatment-related risk factors, previous treatments, such as RT/CRT, causing tissue fibrosis and skin necrosis, can lead to delayed mucosa healing, resulting in PCF. Neck dissection, requiring a longer operating time, could lead to an increased risk of wound infection and PCF formation. In addition, the skill of the surgeon, especially with regard to watertight closure of the pharyngeal wall, could represent a risk factor for PCF formation.

The “fistula zero protocol” was introduced to minimize the rate of PCF formation after TL, based on information from literature data. Many studies have analyzed the usefulness of the salivary bypass tube in preventing PCF formation, but the heterogeneity of patients included in those studies did not allow a meaningful meta-analysis to support strong evidence in clinical practice (11). The salivary bypass tube, placed for the first time in 1978 by Montgomery (18) to bridge the gap between the pharyngostome and esophagostome after laryngoesophagectomy, is recommended in high-risk patients to prevent PCF formation after total laryngectomy and to prevent stenosis of the cervical esophagus and tracheoesophageal fistula (11, 19).

More recently, Gilardi et al. precisely described how to manage the positioning of a salivary bypass tube with the help of a Cuffed-Reinforced Oral/Nasal Tracheal Tube, to prevent or treat some complications of laryngeal and hypopharyngeal surgery (20). Moreover, many authors have previously endorsed the application of a salivary pharyngeal tube, often in association with reconstructive surgery, to help wound healing in patients who developed PCF after laryngeal surgery (21–24).

Concerning the present study, the calyx-shaped Har-El pharyngeal tube allows better adaptability to the pharynx, allowing an efficient collection of saliva inside the tube, therefore reducing its spread over the pharyngeal suture.

On the other hand, based on literature data, closure of the pharyngeal wall could represent a risk factor for PCF formation. Several studies have demonstrated that a mechanical pharyngeal suture could represent an advantage in patients undergoing total laryngectomy, mainly for the reduction in PCF formation (8.7% with an absolute risk reduction of 15%). Nevertheless, the benefits offered by stapler-assisted closure could not be definitively identified among patients who had previously undergone organ-sparing protocols (25).

In the fistula zero protocol, the pharyngeal suture is performed horizontally using a barbed suture. Currently, three types of barbed suture are commercially available: the Quill Self-Retaining System (SRS) (B. Braun Medical Ltd, Sheffield, UK), the bidirectional barbed suture (Angiotech, Vancouver, BC, Canada) and the V-Loc unidirectional barbed suture (V-lock suture; Covidien™, Mansfield, VA, USA) (26–28). The barbed stitches allow for a continuous suture, without tension, reducing the possibility of knots slipping, secondary dehiscence to knot breakage, extrusion or suture splitting and necrosis caused by knot compression on tissues. Furthermore, a continuous suture without knots gives a better seal against liquids, reducing the potential infiltration of saliva between tissues of different thickness and consistency. Three stitches are normally used to complete the tension-free suture.

The third key point of our project is using a pedicled flap to reinforce the pharyngeal suture in pre-treated patients (radiotherapy with or without concomitant chemotherapy). The onlay of a pedicled/free flap in pre-treated patients is now widely supported in the literature, because of the radiation-induced microvascular injury that leads to hypovascular, hypocellular, and hypoxic tissue. Many authors suggest that reinforcing the pharyngeal suture with well-vascularized tissue will help to reduce the incidence of PCF formation in these patients (5, 29, 30). The pectoralis major myofascial flap is one of the most reliable flaps, but many other reconstructive options are described (supraclavicular artery island flap, fasciocutaneous free flaps, mammary artery perforator propeller flap, latissimus dorsi flaps, and facial artery-based cutaneous island flap) (21, 22, 31–34).

In our practice, a pectoralis major myofascial flap was sutured over the pharyngeal suture, without compressing it. The systematic and rigorous adoption of our protocol allowed us to observe excellent results in terms of minimal complications (1.3%), especially when comparing pre-treated (1.3%) and untreated (3.9%) patients, showing similar low rates of complications, also stratifying the patients on the basis of patient- and disease-related risk factors.

The management of PCF has a huge economic impact. Parikh et al. estimated that 57% of patients who develop a fistula require surgical revision. They reported postoperative complications in 22% of patients, with a final cost, which included hospitalization and surgical procedures, of about $58 000 for each fistula (35).

The results of this study seem to be encouraging from both a clinical and economic perspective. In our cohort, the mean hospitalization time was 18 days (range 12–40 days). Hospitalization was longer (21 days) in pre-treated patients (reconstruction; donor-site seroma occurred in four patients and was managed with compression dressing) and shorter (16 days) for patients who did not undergo reconstruction. Higher surgery-related costs (mean € 550 for each procedure) are balanced by a reduction in the length of hospitalization and the absence of delayed adjuvant therapy and oral feeding.

The strengths of this study are represented by the relatively large series and uniformity of surgical procedures. The main limitation of the study is the lack of a control group of patients not treated with this protocol. The preliminary results obtained in this study encourage us to propose a multi-institutional perspective study to validate the usefulness of the Har-El salivary bypass tube in preventing PCF after TL.

## Conclusion

Sustainability of medical care, especially in oncology, is a delicate and debated topic, especially in a world where comorbidities increase with increasing age of the population. More standardized procedures are required, and total laryngectomy represents an excellent model from this point of view.

“Fistula zero” is undoubtedly an ambitious project (there is no surgery without complications) but not an unapproachable strategy. Thanks to a careful attitude and a meticulous approach, the project achieved very low rates of pharyngocutaneous fistula and small blind fistula formation.

The results of this study were obtained by rigid adherence to the protocol and uniformity of surgical procedure and suggest that, with a small increase in surgical costs, it is possible to reduce overall costs for PCF management.

## Data Availability Statement

The original contributions presented in the study are included in the article/supplementary material. Further inquiries can be directed to the corresponding author.

## Ethics Statement

Ethical review and approval was not required for the study on human participants in accordance with the local legislation and institutional requirements. The patients/participants provided their written informed consent to participate in this study.

## Author Contributions

EC, conception and design of the study, surgeon, writing and editing the manuscript. GA, data collection. AS, medical statistician who performed statistical analysis. GS, conception and design of the study, surgeon, editing the manuscript. All authors contributed to the article and approved the submitted version.

## Funding

This research was funded by: Regione Piemonte, Progetto A Funzione (years 2019-2021); FPRC 5x1000 2016 Ministero della Salute Progetto ARDITE - BioHeNeC; Fondi Ricerca Corrente 2021, Ministero della Salute.

## Conflict of Interest

The authors declare that the research was conducted in the absence of any commercial or financial relationships that could be construed as a potential conflict of interest.

## Abbreviations

COPD, chronic obstructive pulmonary disease; PCF, pharyngocutaneous fistula; TL, total laryngectomy; NGT, naso-gastric tube; ND, neck dissection; TLM, transoral laser microsurgery; OPHL, open partial horizontal laryngectomy.

## References

[1] Thawley SE. Complications of Combined Radiation Therapy and Surgery for Carcinoma of the Larynx and Inferior Hypopharynx. Laryngoscope (1981) 91(5):677–700. 10.1288/00005537-198105000-00001 7231019

[2] Bresson K Rasmussen H Rasmussen PA. Pharyngo-Cutaneous Fistulae in Totally Laryngectomized Patients. J Laryngol Otol (1974) 88(9):835–42. 10.1017/s0022215100079433 4214887

[3] Sayles M Grant DG. Preventing Pharyngo-Cutaneous Fistula in Total Laryngectomy: A Systematic Review and Meta-Analysis. Laryngoscope (2014) 124(5):1150–63. 10.1002/lary.24448 24122657

[4] Süslü N Senirli RT Günaydın RÖ Özer S Karakaya J Hoşal AŞ. Pharyngocutaneous Fistula After Salvage Laryngectomy. Acta Otolaryngol (2015) 135(6):615–21. 10.3109/00016489.2015.1009639 25762119

[5] Sittitrai P Srivanitchapoom C Reunmakkaew D. Prevention of Pharyngocutaneous Fistula in Salvage Total Laryngectomy: Role of the Pectoralis Major Flap and Peri-Operative Management. J Laryngol Otol (2018) 132(3):246–51. 10.1017/S0022215118000178 29512475

[6] Lemaire E Schultz P Vergez S Debry C Sarini J Vairel B. Risk Factors for Pharyngocutaneous Fistula After Total Pharyngolaryngectomy [Published Online Ahead of Print, 2020 Feb 25]. Ear Nose Throat J (2020) 1–7. 10.1177/0145561319901035 145561319901035. 32098492

[7] Wang M Xun Y Wang K Lu L Yu A Guan B. Risk Factors of Pharyngocutaneous Fistula After Total Laryngectomy: A Systematic Review and Meta-Analysis. Eur Arch Otorhinolaryngol (2020) 277(2):585–99. 10.1007/s00405-019-05718-9 31712878

[8] Minni A Ralli M Di Cianni S Cialente F Candelori F Colizza A. Montgomery Salivary Bypass Tube in Head and Neck Cancer: The Experience of Our Otolaryngology Clinic. Ear Nose Throat J (2020) 12:1–5. 10.1177/0145561320961754 145561320961754. 33044843

[9] Piazza C Paderno A Del Bon F Grammatica A Montalto N Bresciani L. Fascio-Cutaneous-Free Flaps As Primary Reconstruction in Salvage Total Laryngectomy. Eur Arch Otorhinolaryngol (2021) 278(1):219–26. 10.1007/s00405-020-06137-x 32583182

[10] Hone RWA Rahman E Wong G Annan Y Alexander V Al-Lami A. Do Salivary Bypass Tubes Lower the Incidence of Pharyngocutaneous Fistula Following Total Laryngectomy? A Retrospective Analysis of Predictive Factors Using Multivariate Analysis. Eur Arch Otorhinolaryngol (2017) 274(4):1983–91. 10.1007/s00405-016-4391-9 PMC5340845 28011997

[11] Kamhieh Y Fox H Hallett E Berry S. Routine Use of Salivary Bypass Tubes in Laryngectomy Patients: Systematic Review. J Laryngol Otol (2018) 132(5):380–4. 10.1017/S0022215118000154 29444718

[12] Bang D Piccirillo JF Littenberg B Johnston A. The Adult Comorbidity Evaluation-27 (Ace-27) Test: A New Comorbidity Index for Patients With Cancer, in: 36th Annual Meeting of American Society of Clinical Oncology, May 20, 2000. New Orleans, LA.

[13] Redaelli de Zinis LO Ferrari L Tomenzoli D Premoli G Parrinello G Nicolai P. Postlaryngectomy Pharyngocutaneous Fistula: Incidence, Predisposing Factors, and Therapy. Head Neck (1999) 21(2):131–8. 10.1002/(sici)1097-0347(199903)21:2<131::aid-hed6>3.0.co;2-f 10091981

[14] Paydarfar JA Birkmeyer NJ. Complications in Head and Neck Surgery: A Meta-Analysis of Postlaryngectomy Pharyngocutaneous Fistula. Arch Otolaryngol Head Neck Surg (2006) 132(1):67–72. 10.1001/archotol.132.1.67 16415432

[15] Wulff NB Kristensen CA Andersen E Charabi B Sørensen CH Homøe P. Risk Factors for Postoperative Complications After Total Laryngectomy Following Radiotherapy or Chemoradiation: A 10-Year Retrospective Longitudinal Study in Eastern Denmark. Clin Otolaryngol (2015) 40(6):662–71. 10.1111/coa.12443 25891761

[16] Busoni M Deganello A Gallo O. Pharyngocutaneous Fistula Following Total Laryngectomy: Analysis of Risk Factors, Prognosis and Treatment Modalities. Acta Otorhinolaryngol Ital (2015) 35(6):400–5. 10.14639/0392-100X-626 PMC4755046 26900245

[17] Lebo NL Caulley L Alsaffar H Corsten MJ Johnson-Obaseki S. Peri-Operative Factors Predisposing to Pharyngocutaneous Fistula After Total Laryngectomy: Analysis of a Large Multi-Institutional Patient Cohort. J Otolaryngol Head Neck Surg (2017) 46(1):54. 10.1186/s40463-017-0233-z 28830509 PMC5568352

[18] Montgomery WW. Salivary Bypass Tube. Ann Otol Rhinol Laryngol (1978) 87(2 Pt 1):159–62. 10.1177/000348947808700202 417655

[19] Bondi S Giordano L Limardo P Bussi M. Role of Montgomery Salivary Stent Placement During Pharyngolaryngectomy, to Prevent Pharyngocutaneous Fistula in High-Risk Patients. J Laryngol Otol (2013) 127(1):54–7. 10.1017/S0022215112002502 23164139

[20] Gilardi A Colizza A Minni A de Vincentiis M. A New Montgomery^®^ Salivary Bypass Tube Placement Technique: Report of Procedures Performed on Patients With Tracheoesophageal Fistula or Pharyngoesophageal Stenosis. Ear Nose Throat J (2021) 25:1–3. 10.1177/01455613211006002 1455613211006002. 33764199

[21] Piazza C Bon FD Paderno A Grammatica A Montalto N Taglietti V. Fasciocutaneous Free Flaps for Reconstruction of Hypopharyngeal Defects. Laryngoscope (2017) 127(12):2731–7. 10.1002/lary.26705 28573675

[22] López F Obeso S Camporro D Fueyo A Suárez C Llorente JL. Outcomes Following Pharyngolaryngectomy With Fasciocutaneous Free Flap Reconstruction and Salivary Bypass Tube. Laryngoscope (2013) 123(3):591–6. 10.1002/lary.23695 22951963

[23] Murray DJ Gilbert RW Vesely MJ Novak CB Zaitlin-Gencher S Clark JR. Functional Outcomes and Donor Site Morbidity Following Circumferential Pharyngoesophageal Reconstruction Using an Anterolateral Thigh Flap and Salivary Bypass Tube. Head Neck (2007) 29(2):147–54. 10.1002/hed.20489 17022086

[24] Varvares MA Cheney ML Gliklich RE Boyd JM Goldsmith T Lazor J. Use of the Radial Forearm Fasciocutaneous Free Flap and Montgomery Salivary Bypass Tube for Pharyngoesophageal Reconstruction. Head Neck (2000) 22:463–8. 10.1002/1097-0347(200008)22:5<463::aid-hed4>3.0.co;2-s 10897105

[25] Aires FT Dedivitis RA Castro MAF Bernardo WM Cernea CR Brandão LG. Efficacy of Stapler Pharyngeal Closure After Total Laryngectomy: A Systematic Review. Head Neck (2014) 36(5):739–42. 10.1002/hed.23326 23729357

[26] Crosetti E Caracciolo A Arrigoni G Delmastro E Succo G. Barbed Suture in Oral Cavity Reconstruction: Preliminary Results. Acta Otorhinolaryngol Ital (2019) 39(5):308–15. 10.14639/0392-100X-2130 PMC6843584 30745594

[27] Villa MT White LE Alam M Yoo SS Walton RL. Barbed Sutures: A Review of the Literature. Plast Reconstr Surg (2008) 121(3):102e–8e. 10.1097/01.prs.0000299452.24743.65 18317092

[28] Moya AP. Barbed Sutures in Body Surgery. Aesthet Surg J (2013) 33(3 Suppl):57S–71S. 10.1177/1090820X13499577 24084880

[29] Oosthuizen JC Leonard DS Kinsella JB. The Role of Pectoralis Major Myofascial Flap in Salvage Laryngectomy: A Single Surgeon Experience. Acta Otolaryngol (2012) 132(9):1002–5. 10.3109/00016489.2012.672768 22568602

[30] Patel UA Keni SP. Pectoralis Myofascial Flap During Salvage Laryngectomy Prevents Pharyngocutaneous Fistula. Otolaryngol Head Neck Surg (2009) 141(2):190–5. 10.1016/j.otohns.2009.03.024 19643250

[31] Emerick KS Herr MA Deschler DG. Supraclavicular Flap Reconstruction Following Total Laryngectomy. Laryngoscope (2014) 124(8):1777–82. 10.1002/lary.24530 24431133

[32] Pabiszczak M Banaszewski J Pastusiak T Szyfter W. Supraclavicular Artery Pedicled Flap in Reconstruction of Pharyngocutaneous Fistulas After Total Laryngectomy. Otolaryngol Pol (2015) 69(2):9–13. 10.5604/00306657.1147032 26224224

[33] lmadori G De Corso E Visconti G Almadori A Di Cintio G Mele DA. Impact of Internal Mammary Artery Perforator Propeller Flap in Neck Resurfacing and Fistula Closure After Salvage Larynx Cancer Surgery: Our Experience. Head Neck (2019) 41(11):3788–97. 10.1002/hed.25903 31397524

[34] Gupta DK Chugh R Singh SK Pati S. Use of the Facial Artery-Based Cutaneous Island Flap (Melo-Labial Flap) for Reconstruction of the Neopharynx Following Total Laryngectomy: A Novel Technique. BMJ Case Rep (2019) 12(8):e230712. 10.1136/bcr-2019-230712 PMC6685414 31383687

[35] Parikh SR Irish JC Curran AJ Gullane PJ Brown DH Rotstein LE. Pharyngocutaneous Fistulae in Laryngectomy Patients: The Toronto Hospital Experience. J Otolaryngol (1998) 27(3):136–40. 9664242

